# Remotely Supervised Home-Based Intensive Exercise Intervention to Improve Balance, Functional Mobility, and Physical Activity in Survivors of Moderate or Severe Traumatic Brain Injury: Protocol for a Mixed Methods Study

**DOI:** 10.2196/14867

**Published:** 2019-10-09

**Authors:** Jennifer O'Neil, Mary Egan, Shawn Marshall, Martin Bilodeau, Luc Pelletier, Heidi Sveistrup

**Affiliations:** 1 School of Rehabilitation Sciences, Faculty of Health Sciences University of Ottawa Ottawa, ON Canada; 2 Bruyère Research Institute Ottawa, ON Canada; 3 Physical Medicine and Rehabilitation Faculty of Medicine University of Ottawa Ottawa, ON Canada; 4 School of Psychology, Faculty of Social Sciences University of Ottawa Ottawa, ON Canada

**Keywords:** telerehabilitation, physical activity, traumatic brain injury, home-based exercises, accessibility

## Abstract

**Background:**

Traumatic brain injury (TBI) may impact an individual physically, cognitively, socially, and emotionally. Poor balance, reduced mobility, and low daily physical activity often will require ongoing physical rehabilitation intervention. However, face-to-face specialized physiotherapy is not always accessible for individuals living in rural settings.

**Objective:**

We will answer four questions: (1) What is the feasibility of a remotely supervised, home-based, intensive exercise intervention with survivors of moderate and severe TBI? (2) Does the frequency of remote supervision have an impact on the feasibility of completing a home-based intensive exercise program? (3) Does the frequency of remote supervision impact balance, functional mobility, and physical activity? (4) What is the lived experience of remote supervision for both survivors and caregivers?

**Methods:**

Four participants will complete two intensive, 4-week (five days per week) home-based exercise interventions remotely supervised via synchronous videoconference. Each exercise intervention will have a goal of 160 to 300 repetitions or 60 minutes of tailored exercises to promote neuroplasticity and be defined as an intensive home-based exercise intervention. An alternating single-subject design will allow for the comparison between two frequencies of remote supervision, once weekly and five times weekly. Daily repeated outcome measures, pre- and postintervention outcome measures, and 1-month follow-up outcome measures will be collected to explore the effect on feasibility and physical variables. Daily outcome measures include step count and Five Times Sit-to-Stand test. Pre-post measures include assessment of quiet stance and the Community Balance and Mobility Scale. A semistructured interview will be completed at the end of each intervention segment to document the lived experience of both survivors and their study partners. Finally, five questionnaires will be used to understand the overall experience: the Mayo-Portland Adaptability Inventory-4 Participation Index, Satisfaction With Life Scale, Fall Efficacy Scale-International, Interpersonal Behavior Questionnaire, and System Usability Scale. Data will be analyzed following traditional single-subject methods of analysis.

**Results:**

Ethics approval was received from both the Bruyère Research Institute and University of Ottawa review boards in March 2019. Recruitment is underway.

**Conclusions:**

The proposed intervention is complex in nature due to the involvement of multiple technology sources and the inclusion of a complex dyad (survivors and caregivers) in a community setting. This type of research is timely given that alternative methods of physical intervention delivery are needed to facilitate gains in balance, mobility, physical activity among TBI survivors with limited access to clinical care, and the quality of the patients’ experience.

**International Registered Report Identifier (IRRID):**

PRR1-10.2196/14867

## Introduction

### Background

A traumatic brain injury (TBI) can impact an individual physically, cognitively, socially, and emotionally, as described by the Ontario Neurotrauma Foundation [1]. These impairments can become chronic, influencing the quality of life of an individual throughout their life span. One-third of military personnel who survived a moderate or severe TBI have continued issues with activities of daily living even up to 14 years postinjury [2,3]. Physical deficits can present as poor balance [4], reduced mobility, and low daily physical activity [5]. These impairments may lead to decreased social participation [6], increased fear of falling [7], and may invoke social isolation [8].

Physical rehabilitation professionals, such as physiotherapists, can influence the recovery of these deficits [9,10], which may positively influence daily function [7]. Traditional face-to-face outpatient physiotherapy sessions are usually completed in-clinic, in-person with a therapist; however, this option is not always accessible to the affected individual. Inequities can be associated with geography, financial barriers, or acceptability [11]. A lack of available knowledgeable professionals in rural communities, increased travel cost and travel time to urban centers, and injury characteristics are examples of barriers for survivors of moderate or severe TBI living in rural communities [7,12,13].

For these reasons, it is crucial to investigate alternative methods of treatment delivery that could impact the accessibility of specialized physiotherapy services. Synchronous remote supervision refers to a form of remote supervision, such as videoconferencing, which allows patient information to be received and recorded in real time by the supervising therapist, and for the therapist to provide immediate feedback [14,15]. Previous studies have assessed the usability of telerehabilitation for the neurologically impaired population and found it to be “as feasible as usual care for upper extremity function in stroke, TBI, and MS [multiple sclerosis]” [16]. Balance interventions delivered remotely by telerehabilitation were also shown to be effective for people with neurological diagnoses [17,18].

To facilitate neuroplasticity, increasing patients’ daily physical activity participation and the number of exercise repetitions they perform are key factors [19], and must be included in any home-based exercise program. Most effective exercise programs for balance, functional mobility, and physical activity for TBI survivors range between 16 and 20 sessions completed in 4 to 6 weeks [10,20-23]. These exercise programs were delivered face-to-face or as a home program without remote supervision. Therefore, we are still uncertain of the effect of frequency for remotely delivered programs.

Exploring the effect frequency of supervision has on different outcomes is needed to develop accessible and effective interventions for this population. A recent study by Lacroix et al [24] explored the dose-response relationship between static balance outcomes and supervision in older adults. They found increased effects with supervision compared with no supervision. They also showed that a low number of supervised sessions (mean 6.7 sessions) was enough to have a positive effect on outcomes, but not as much as full supervision (mean 30 sessions). This indicates that having supervised sessions is important for improving motor skills. However, we are still uncertain of the specific amount of supervision needed to have an impact on physical impairments while remaining feasible for survivors. Minimal research has been done in this area for TBI survivors. It is feasible for this population to independently complete a community exercise program [25]; however, the optimal frequency of remote supervision that will positively impact physical outcome measures is unclear.

### Objectives

This study will determine the feasibility and effectiveness of two frequencies of remote supervision for a home-based intensive exercise intervention (daily and weekly supervision) for military or veteran survivors of moderate or severe TBI. Four main questions will be investigated:

1. What is the feasibility of a remotely supervised home-based intensive exercise intervention with survivors of moderate and severe TBI?
2. Does the frequency of remote supervision have an impact on the feasibility of completing a home-based intensive exercise program?
3. Does the frequency of remote supervision impact balance, functional mobility, and physical activity?
4. What is the lived experience of remote supervision for both survivors and caregivers?

We hypothesize that a remotely delivered exercise program targeting balance, mobility, and increased physical activity will be feasible and that frequency of supervision will have an impact on the effectiveness of the intervention. We also hypothesize that the dyad (survivor and caregiver) will report a satisfactory experience.

## Methods

### Overview

**Figure 1:**
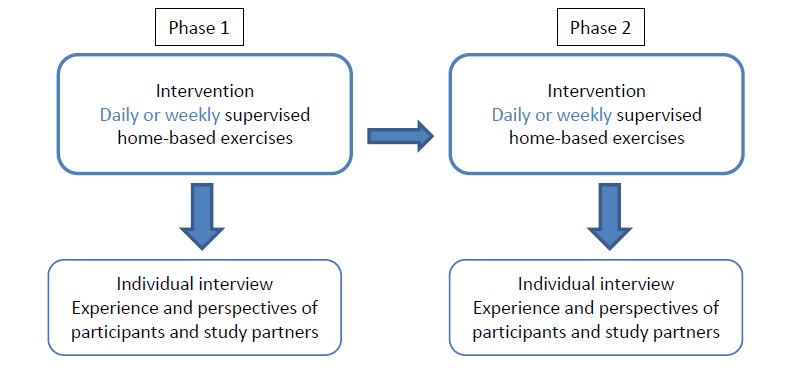
Sequential explanatory approach demonstrating the phases of the remotely supervised home-based interventions differing in supervision frequencies. A crossover design will be used; two participants will begin with daily supervision and two participants will begin with weekly supervision.

This study will follow a sequential explanatory mixed methods intervention approach (Figure 1) [26]. Qualitative observations collected via semistructured interviews will help interpret and better qualify information stemming from the quantitative data.

A single-subject design is often used to compare different interventions in clinical settings [27-29] and provides a way to explore the feasibility and potential effectiveness in the early stages of investigating a new intervention. An alternating single-subject design will allow for comparison between the two frequencies of remote supervision. The phase sequence determined a priori will include baseline (6 data points), intervention 1 for 4 weeks (20 data points), a 4-week washout period, intervention 2 for 4 weeks (20 data points), a postintervention phase (3 data points), and a 1-month follow-up phase (1 data point). A 4-week washout period will be added to mitigate the effects of the first intervention on the second intervention. During this period, the participants will be asked not to perform any of the specific exercises from the intervention program. Replication across four participants will help establish if there is a causal relationship.

### Participants

Four survivors of moderate to severe TBI aged 18 to 64 years and their respective study partners will be recruited from the community via recruitment posters and videos. Participants will be eligible if they report a diagnosis of a moderate or severe TBI a year or more ago. This diagnostic can be defined as a score of 12 or less on the Glasgow Coma Scale [30], time spent in an in-patient unit, loss of consciousness with trauma, or presence of posttraumatic amnesia. To be included, participants must speak English or French and live in the community (not in the hospital or long-term care facilities). The ability to stand independently for a minimum of 2 minutes will be required. Participants will be asked to confirm this on the screening call and will be assessed for 2 minutes stance at the first assessment session. In addition, the TBI survivors will need a consenting study partner. The relationship between study partner and participant can be of a personal nature, such as caregiver, family member, personal support worker, or friend willing to participate with the survivor for the entire study.

Potential participants will be excluded if they report symptoms of vertigo, measured by questions from the Dizziness Handicap Inventory [31], or are unable to use the videoconferencing system.

### Exercise Intervention

Interventions will take place in the participants’ homes. Five face-to-face visits at the Bruyère Research Institute in Ottawa, Canada, will take place for assessment purposes. All exercise interventions will be completed remotely.

A familiarization session during the first face-to-face visit at the Bruyère Research Institute will mark the first day of the baseline phase. The participant and study partner will be trained on the use of the activity tracking device and the videoconference platform. Education on safety precautions during the home-based exercise intervention session will be provided (ie, wearing proper shoes, removing clutter, having enough space to move, having a chair near to take breaks when required, instructions in case of presence of dizziness, injuries, or falls). The exercise intervention objectives will be discussed with the dyad, such as “the goal of this exercise intervention is to increase your physical activity levels, improve your balance and mobility by spending 30 to 60 minutes per day exercising. The goal is to reach a minimum amount of repetitions per session ranging between 160 and 300 repetitions.” Questions related to individual abilities, goals, motivation, and adherence strategies will be asked to tailor the intervention to each participant. Collaboration with the study partner will take place to tailor the daily diary log and facilitate its completion. A participant manual, including daily exercises, safety precautions, and technology information, will be provided to each study dyad. TBI survivors assisted by the study partner will be asked to complete a daily log including step count and the Five Times Sit-to-Stand (FTSTS) test [32,33].

The four participants will complete two intensive, 4-week (five days per week), home-based exercise interventions remotely supervised via synchronous videoconference. Participants will be supervised via videoconferencing daily or weekly for the entire proposed intervention period. During the weekly remote supervision intervention, remote supervision will be provided on Mondays; for the remaining days of the week, participants will complete the intervention aided by their study partner, but unsupervised by the researcher. Each participant will be emailed a link to start the videoconference intervention 15 minutes before starting the intervention, and the study partner will facilitate connection with the therapist. The therapist in charge will collaborate with the dyad to ensure proper positioning of the camera and safe environment.

A trained and registered physiotherapist with over 10 years of experience in rehabilitation of TBI will provide all remote supervision sessions via the Ontario Telemedicine Network. Each participant will be asked to complete the exercise intervention at the same time of the day. Individual exercise sessions will be structured and tailored to each participant as defined in the familiarization session. The exercise intervention will consist of two to four exercises targeting essential components of dynamic balance, weight shifting, reaction time, and postural control (Table 1). As an example, sit-to-stand, side stepping, lunges, squats, tandem walking, and single-leg stance could be included in the exercise intervention (Multimedia Appendix 1). These specific exercises were chosen because they were included in previous programs targeting dynamic balance in TBI survivors [5,10,20-23]. Exercises will be completed 1 minute at a time, for a total of 10 minutes per specific exercise. When possible (ie, with the sit-to-stand exercise), the number of repetitions of the exercises will be recorded by the therapist or study partner at the end of each minute and entered in the daily log. If a participant cannot complete the exercises as planned, the physiotherapist will provide options ahead of time to modify the exercises to enable the participant to be successful and reach the desired intensity; for example, modifying the height of a stepping block.

**Table 1:** Tailoring of intervention in collaboration with each dyad (participant and study partner).

Intervention parameters	Tailoring options
Numbers of sessions	20 sessions, 5 days per week, for 4 weeks
Length of each exercise session	Options for each participant: (1) 60 minutes maximum and a minimum of 160 repetitions; (2) reaching 160-300 repetitions
Number of exercises	Tailored for each participant: 2 to 4
Goal of exercises	Targeting balance, mobility, and gait components
Exercise options	(1) Sit-to-stand from a chair, stool, couch; (2) step-up, side steps, high knees, squats, short lunges; (3) standing still, feet together, with arm movements; (4) walking between parallel lines 14-inches apart, walking forward placing foot on lines, walking sideways

### Technology

Participants will be provided with a computer for the duration of the study to have access to the remote supervision. The Ontario Telemedicine Network will allow for secure and confidential remote supervision to be completed via videoconference. This platform adheres to all personal health information policies. A link will be emailed to the participant before each session, and a one-click process will facilitate access to the remote supervision. Audio and video will be recorded using screen capture software (Bandicam software), eliminating the use of cloud-based storage.

An individual activity tracking device dashboard will be set up for each participant on the computer. The activity tracking device information collected daily will be synchronized, and the research team will be able to access the dashboard remotely.

### Feasibility Measures

Measures of feasibility will be recorded throughout the study. As per Thabane et al [34], four different feasibility factors will be measured: (1) process (adherence rate, retention rate, recruitment rate), (2) resources (length of the intervention, equipment, capacity of transportation for assessments), (3) management (data management), and (4) scientific aspects (safety, dose; Table 2).

### Outcomes Measures

Daily repeated measures, pre-post intervention measures, and 1-month follow-up measures will be recorded (Table 3).

**Table 2:** Measures of feasibility.

Feasibility factors		Measurements
**Process**		
	Adherence rate	Number sessions completed
	Retention	Number of participants recruited, number of participants completing study
	Recruitment	Severity, transportation issues, availability of study partner, time
**Resources**		
	Length of intervention	Minutes of interventions
	Equipment	Reported issues, cost
	Transportation capacity	For five face-to-face visits
**Management**		
	Data management	Time spent on data collection and analysis, therapist time on remote supervision
**Scientific**		
	Safety	Adverse events
	Dose	Number of repetitions, number of sessions

**Table 3:** Outcome measures and data collection protocol.

Outcome measures		Baseline	Daily remote supervision^a^		Weekly remote supervision^a^			1-month follow-up
			During exercise program	Posttest	Pretest	During exercise program	Posttest	
**Feasibility**								
	Adherence	x	x	x	x	x	x	x
	Adverse events	x	x	x	x	x	x	x
	Retention	x						x
	Recruitment	x						
	Equipment	x	x	x	x	x	x	x
	Transportation	x		x	x		x	x
	Length of intervention		x			x		
	Data management	x	x	x	x	x	x	x
	Dosage		x			x		
**Repeated measures**								
	Step count	x	x	x	x	x	x	x
	Five Times Sit-to-Stand test	x	x^b^	x	x	x^b^	x	x
**Pre-post measures**								
	Quiet stance (two conditions)	x		x	x		x	x
	Community Balance & Mobility Scale	x		x	x		x	x
**Pre-post self-reported**								
	Mayo-Portland Participation Index	x		x	x		x	
	Fall Efficacy Scale-International	x		x	x		x	
	Satisfaction with Life Scale	x		x	x		x	
	IBQ^c^ and IBQ-Self	x		x			x	
	System Usability Scale			x				

^a^These two interventions will be alternating for participants 1 and 2 and participants 3 and 4.

^b^On Mondays with a therapist.

^c^IBQ: Interpersonal Behavior Questionnaire.

#### Primary Measures: Daily Repeated Measures

Physical activity will be measured by recording step count with an activity tracking device placed at the ankle throughout the entire study [35]. Step count will be recorded every day between intervention sessions to document the effect of the intervention on the amount of physical activity, and during the activity sessions to monitor the number of stepping repetitions.

Functional mobility will be recorded daily with the completion of the FTSTS test. The FTSTS test measures lower extremity muscle strength and transitional movements [33], which contribute to poor balance when decreased [36]. For both supervision frequencies, the FTSTS test will be assessed by the therapist and study partner remotely on Mondays and by the study partner alone during the remaining weekdays.

#### Secondary Measures: Pre-Post Intervention Measures

As part of the five in-person visits, pre-post intervention measurements will be collected within 5 days of intervention completion. Standing balance will be measured pre- and postintervention for each intervention using the Balance Tracking System Inc (BTracks) [37]. Participants will be asked to stand as still as possible with their feet hip-width apart on the balance board and hands placed on their hips. Two conditions of quiet stance will be measured—eyes open and eyes closed—for three trials of 30 seconds for each condition [38]. Mean velocity (cm/s), root mean square in the anteroposterior and mediolateral planes, and center of pressure (COP) area (95% confidence interval ellipse) will be computed from the balance data.

Functional balance will be measured with the Community Balance and Mobility Scale (CB&M) [39,40]. This clinical test will be administered by an experienced physiotherapist blinded to the intervention study phase (eg, daily or weekly supervision) for each frequency of remote supervision.

Three self-reported questionnaires will be collected pre- and postintervention for both frequencies of remote supervision: the Mayo-Portland Participation Index (M2PI) [41], the Satisfaction With Life Scale [42], and the Fall Efficacy Scale-International. The Mayo-Portland Adaptability Inventory-4, which includes the M2PI Participation Index, is increasingly used in TBI research and allows for a comprehensive picture of ability, adjustment, and participation. The M2PI [41] is a participation index in which eight domains assess a person’s issues within different situations on a Likert scale ranging from 1 to 4. This index will be completed to capture data on participation and explore the impact that remotely supervised physical interventions can have on these three physical domains.

The Satisfaction With Life Scale [42] will be added as a measure of intervention effectiveness. This scale has been widely used with individuals who have survived a moderate or severe TBI and will provide insight into the impact of the remote supervision interventions on overall life satisfaction [43].

The Fall Efficacy Scale-International will allow for a subjective assessment of the fear of falling pre- and postintervention [44]. Fear of falling influences the ability to perform rehabilitation programs independently [45] and is directly correlated with balance deficits [7]. A cut-off score of greater than 23 can be defined as presenting with a fear of falling [46].

One novel questionnaire will be administered at three points in time—baseline session, postintervention 1, and postintervention 2—to assess the quality of the interaction between the therapist and patient. The Interpersonal Behavior Questionnaire (IBQ) [47,48] will assess the patient’s perceptions of their therapist’s interpersonal behaviors, and the IBQ-Self will assess the therapists’ perceptions of their own interpersonal behaviors toward their patients. Each participant will complete their respective part of the questionnaire. The IBQ will be coded in six different categories: autonomy support, autonomy thwarting, competence support, competence thwarting, relatedness support, and relatedness thwarting [47,48] to determine if the therapist interpersonal behaviors are need supportive or need thwarting.

Finally, the System Usability Scale will be collected at postintervention 1 to identify the usability of the Ontario Telemedicine Network system to assess whether it is a good fit for this population [49].

#### One-Month Follow-Up Measures

A 1-month follow-up will be scheduled to reassess standing balance, functional mobility, and physical activity. Participants will be contacted and asked to wear the activity tracking device on their ankle for the 3 days before the final face-to-face visit. During the final face-to-face visit, the FTSTS [33] and the CB&M scale assessment [39] will be completed. Postural sway via quiet stance will also be reassessed by completing the same COP measures as in pre-post testing. Finally, step count will be gathered by the dyad over 3 days.

#### Semistructured Interview

As part of the mixed methods design, interviews will be administered with TBI survivors and their study partners separately either face-to-face or via videoconferencing technology at the end of each intervention period (week 4 and week 12). At the beginning of each interview, a brief introduction, confidentiality statement, and description of the research objective will be given to the survivor or the study partner. Each 60-minute, independently completed interview will take place in a private, neutral location [50]. An interview guide will facilitate the probing of topics related to the participant’s overall experience, physical activity, safety, experience with remote supervision, and adherence. All interviews will be audio-recorded and saved, password-protected, and encrypted for confidentiality. Field notes will be used to describe the participants’ behavior, therapist-perceived comfort levels, and nonverbal demeanor, which will help contextualize the interview and situate the experiences within that context.

### Data Analysis

Daily repeated measures data will be analyzed following the four-step single-subject design method of analysis, and pre-post measures for each participant will be descriptively analyzed (Table 4).

**Table 4:** Outcome measures, analysis, and expected changes.

Outcome measures and method of analysis		Expected changes
**Step count**		
	SSD^a^ traditional methods	Increase in step count number
**Five Times Sit-to-Stand**		
	SSD traditional methods	Decrease in time (seconds)
**Quiet stance (center of pressure>)**		
	Descriptive	Velocity: decrease showing an increase in postural stability; root mean square: decrease displacement showing an increase in postural stability; 95% ellipse: decrease displacement showing an increase in postural stability
**Community Balance & Mobility Scale**		
	Descriptive and MDC (7.5)	Increase in total points by a minimum of 7.5
**Questionnaires pre-post**		
	Descriptive	Fall Efficacy Scale-International: decrease number showing decrease concern or fear of falling (score >23 indicates high concern of falling); Mayo-Portland Participation Index: increased index of participation; Satisfaction With Life Scale: increased satisfaction
**Questionnaires**		
	Descriptive	IBQ^b^/IBQ-Self: enhanced communication and self-efficacy between all groups
**Feasibility**		
	Descriptive, feasibility, and process	Adherence: increase in completion of session 80%; retention rate: 100% retention rate; recruitment rate: 80% recruitment rate
	Descriptive, feasibility, and resources	Length of the intervention, dose, and intensity by interview; equipment using the System Usability Scale: high score showing usability; capacity of transportation for assessments
	Descriptive, feasibility, and management	Data management by therapist field note
	Descriptive, feasibility, and scientific aspects	Safety by adverse events: decrease in adverse events

^a^SSD: single-subject design.

^b^IBQ: Interpersonal Behavior Questionnaire.

#### Analysis for Daily Repeated Measures (Step Count and FTSTS)

In step 1, a visual analysis will be completed by analyzing the trend and identifying stability [51]. A visual comparison of the direction and rate of change as well as variability between all phases (baseline, intervention 1, postintervention 1, washout period, intervention 2, and postintervention 2) [51,52] will be completed.

In step 2, serial dependency will be calculated on all raw data using the lag-1 autocorrelation (r) [46]. The baseline phase for all participants will be more than 5 points; therefore, serial dependency will be assessed on baseline data only [53]. In the case of a statistically significant presence of serial dependency confirmed by the Bartlett test, a first-difference transformation will be calculated before visual and statistical analysis. Once serial dependency is eliminated, a single line graph will be constructed for each participant and daily outcome measure [52].

In step 3, the overall changes between baseline and the first intervention phase and the washout phase and the second intervention phase will be analyzed with the two-standard deviation band method [53,54].

In step 4, an effect size analysis will be completed to supplement visual analysis. The effect size will be calculated by the standardized mean difference of all points proposed by Gage and Lewis [55] and Olive and Smith [56].

#### Analysis for Pre-Post Measures

All feasibility measures, CB&M scale, COP measures, and self-reported questionnaires will be analyzed descriptively. Mean and standard deviation will allow for the interpretation of intervention effects on outcome measures (Table 3). Clinical significance will also be considered using the CB&M scale, which is 7.5 points for minimal detectable change [39].

To quantify feasibility, a priori cut-offs were defined based on a similar study [57]. The process factors will be quantified as feasible if 80% of the sessions are completed and 100% retention is achieved; dosage factor will be feasible if 80% of sessions achieve 160 to 300 repetitions.

#### Analysis of Semistructured Interview

A thematic analysis will be used to organize the data [58]. All semistructured interviews will be fully transcribed to text by the main researcher to allow familiarization with the data. Field notes and audio transcription of each interview will be amalgamated, and the main researcher will become deeply involved with the data before disassembling it into codes. A preestablished framework, the theoretical domain framework [59], will be used to facilitate coding of themes into domains often used in behavioral change and implementation research [52]. If the theoretical domain framework is not suitable for all codes, modifications will be made. As such, general thematic codes will be identified for each transcript and will be collated into a matrix. The matrix will provide a visual representation of all the data to facilitate interpretation. This matrix will be reviewed by a second reviewer, and then interpreted in relation to each question. Patterns between the different participants, within their dyad and between interview one and two, will then be analyzed. This deductive-inductive analysis will provide evidence-based information which could enable a deeper comprehension of the lived experience of remotely supervised home-based interventions.

### Ethics

Ethics approval was obtained after a full review process by the Bruyère Research Institute in January 2019, followed by the University of Ottawa board of ethics in March 2019. As per ethics approval, a four-step process will be followed for recruitment and consenting of each participant and caregiver (dyad): recruitment sites will be contacted and recruitment material (poster and video) will be provided, a verbal consent before screening potential participants will be completed on initial contact with primary investigator (J O’Neil, a physiotherapist and PhD candidate), screening of potential participants will be conducted by the primary investigator, and for eligible participants, consent forms will be signed and participants will be given a code to secure confidentiality.

## Results

Recruitment is currently underway. Community physiotherapy clinics, military-associated health care professionals, and brain injury associations have received a recruitment site letter, poster, and video. This study is expected to be completed by December 2020.

## Discussion

The proposed intervention is complex because of the involvement of multiple technology sources and the inclusion of a complex dyad. Furthermore, the community setting will add another dimension of complexity. However, this type of research is timely because alternative methods of physical intervention delivery are needed to facilitate gains in balance, mobility, and physical activity. Patients who are discharged home and entering the chronic phase of their rehabilitation could benefit from completing an intensive home-based exercise intervention delivered remotely to improve their overall independence. A better understanding of remote supervision and its implementation will allow us to inform future studies around the same constructs.

## Appendix

Multimedia Appendix 1 Example of included exercises in tailored home-based program.

## Abbreviations

CB&M: Community Balance and Mobility Scale
COP: center of pressure
FTSTS: Five Times Sit-to-Stand
IBQ: Interpersonal Behavior Questionnaire
TBI: traumatic brain injury
