# Supplementary material for: Remotely Supervised Home-Based Intensive Exercise Intervention to Improve Balance, Functional Mobility, and Physical Activity in Survivors of Moderate or Severe Traumatic Brain Injury: Protocol for a Mixed Methods Study
Source: JMIR Res Protoc. 2019 Oct 9;8(10):e14867. doi: 10.2196/14867 (PMC6812480; doi:10.2196/14867)

Step-up, High knees,

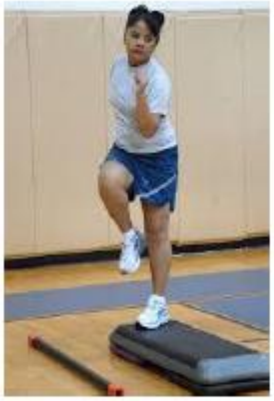

✓ Side steps,

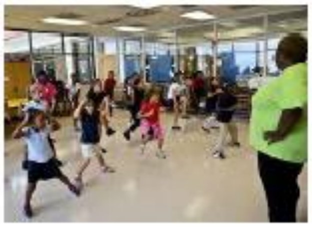

✓ Squats,

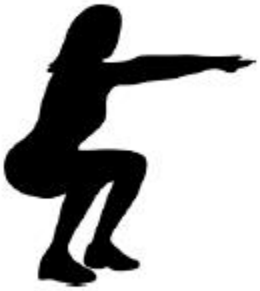

Standing still feet together with arm movements,

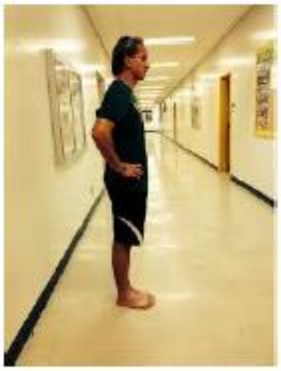

Standing on one leg,

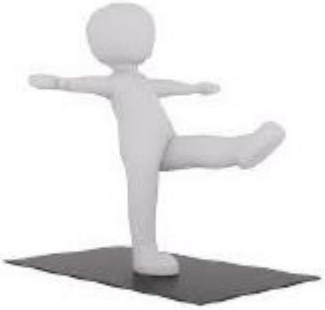

Walking between parallel lines 14 inch apart,

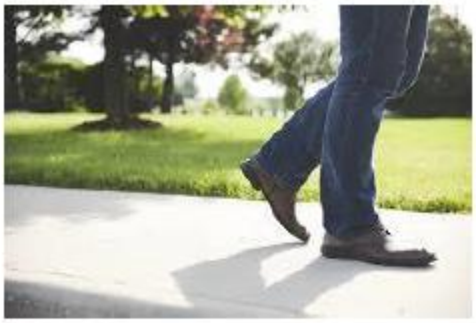

Sit to stand from chair or stool or couch,

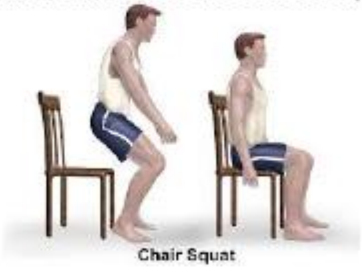

Short lunges,

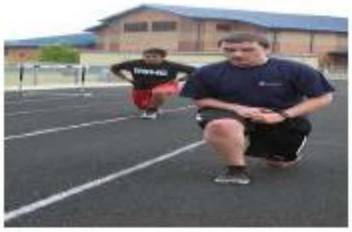

Four square stepping

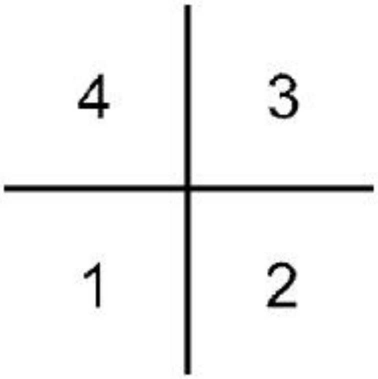

Walk backwards-sideways,

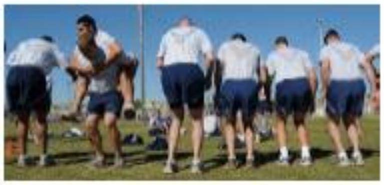

Walk on toes,

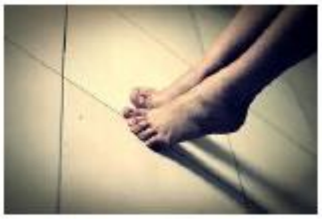

Stepping on star pattern or cross,

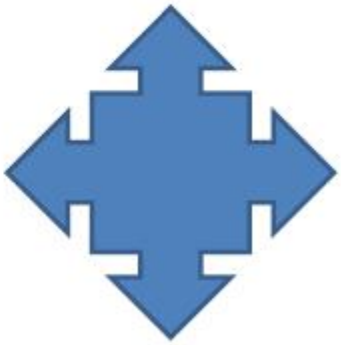

Supplement: Multimedia Appendix 1 [file resprot_v8i9e14867_app1.pdf]
